# Anergic Pulmonary Tuberculosis Is Associated with Contraction of the Vd2+T Cell Population, Apoptosis and Enhanced Inhibitory Cytokine Production

**DOI:** 10.1371/journal.pone.0071245

**Published:** 2013-08-02

**Authors:** Liping Yan, Haiyan Cui, Heping Xiao, Qing Zhang

**Affiliations:** Department of Tuberculosis, Shanghai Pulmonary Hospital, Tongji University School of Medicine, Shanghai, China; Fundació Institut d’Investigació en Ciències de la Salut Germans Trias i Pujol. Universitat Autònoma de Barcelona. CIBERES, Spain

## Abstract

**Objective:**

To study the association of anergic pulmonary tuberculosis with Vδ2^+^ T cells and related cytokine levels.

**Methods:**

82 pulmonary tuberculosis patients were divided into two groups according to their purified protein derivative tuberculin skin test (TST) results: 39 with TST-negative anergic pulmonary tuberculosis and 43 with TST-positive pulmonary tuberculosis, while 40 healthy volunteers were used as control. Based on chest X-ray results, the tuberculosis lesions were scored according to their severity, with a score of ≤ 2.5 ranking as mild, 2.5-6 as moderate and ≥ 6 as severe. The Vδ2^+^ T cell percentage and their expression levels of the apoptosis-related membrane surface molecule FasL in peripheral blood and bronchoalveolar lavage fluids (BALF) were analyzed by flow cytometry, while IL-2, IL-4, IL-6 and IL-10 cytokine and γ-interferon (γ-IFN) concentrations in peripheral blood were determined by ELISA.

**Results:**

Most of the patients with chest X-ray lesion scores higher than 6 belonged to the anergic tuberculosis group (P<0.05). Anergic pulmonary tuberculosis patients displayed reduced peripheral blood Vδ2^+^ T cell counts (P<0.05) and higher FasL expression in peripheral blood Vδ2 ^+^ T cells (P <0.05). The Vδ2^+^ T cell percentages in the BALF of all tuberculosis patients were lower than in their peripheral blood (P <0.05), and IL-4 and IL-10 concentrations in peripheral blood of anergic tuberculosis patients were higher than in TST-positive tuberculosis patients and healthy controls (P <0.05).

**Conclusion:**

Anergic pulmonary tuberculosis is accompanied by reduced Vδ2^+^ T cell percentage, and elevated Vδ2^+^ T cell FasL expression as well as enhanced IL-4 and IL-10 levels in peripheral blood.

## Introduction

Tuberculosis has the greatest mortality rate among all infectious diseases, which is mainly due to the current lack of effective protective vaccines and incomplete understanding of the mechanisms by which *M. tuberculosis* escapes immune surveillance [1,2]. From an immunological point of view, tuberculosis can be classified into several subtypes. One subtype is anergic tuberculosis, with negative TST results; more accurately, anergic tuberculosis, which accounts for about 15% of tuberculosis cases, refers to a disease that is negative for tuberculin purified protein derivative skin tests without a concomitant immunodeficiency disease. These patients often do not display granuloma formation, yet have severe atypical clinical manifestations [3]. It is quite difficult to diagnose and treat anergic tuberculosis patients since their TST is always negative and there are very few reports on clinical features and immunological mechanisms related to anergic tuberculosis. Adaptive immunity against *M. tuberculosis* depends in general on CD4 T cells, but γδ T cells, which account for 1-5% of all peripheral blood T cells [4,5], also play an important role. γδ T cells are not only representatives of early innate immune cells, but also have characteristics of adaptive immune cells. They can recognize pathogens with different types of molecule patterns and regulate the immune responses by participating in immune surveillance, cell migration and activation, as well as tissue repair. γδ T cells constitute a subtype of T cells and are referred to as "non-traditional" T cells [6]. In particular, Vγ9Vδ2 (Vδ2) T cells, a subset of the γδ T cell population, play a unique role in host defense against tuberculosis. Some studies suggest that due to the activation of Vδ2^+^ T cells by phospho-antigens of *M. tuberculosis*, the percentage of these T cells increases in all tuberculosis patients [7]. In addition, peripheral blood mononuclear cells and alveolar macrophages, as antigen presenting cells (APCs), can provide co-stimulatory signals for Vδ2^+^ T cells in tuberculosis foci, which ultimately induce an increase in the cell number, cytokine secretion and cytolytic activity of Vδ2^+^ T cells [8], thus inhibiting the growth of *M. tuberculosis* as well as stimulating the generation of memory immune cells. Another study showed that the amount of Vδ2^+^ T cells in the peripheral blood of anergic tuberculosis patients is significantly decreased [3], mainly due to apoptosis and redistribution of these T cells. Fas and its ligand, FasL, are apoptosis membrane surface molecules and the Fas/FasL pathway has been shown to be associated with apoptosis in γδT cells [9]. Other studies showed that Vδ2^+^ T cells can regulate the immune response by secreting cytokines with different functions [10–12], which contribute to the formation of anergic tuberculosis. The present study sought to further explore associations of anergic tuberculosis with Vδ2^+^ T cell percentages and serum concentrations of related cytokines in order to elucidate factors affecting immunological damage and protection, and to further characterize anti-tuberculosis defense mechanisms, thereby providing the basis for optimized chemotherapy regimens and immunological therapies as well as for designing new vaccines against tuberculosis [13,14].

## Patients and Methods

### Patients

All tuberculosis patients included in this study were collected from Shanghai Pulmonary Hospital between January 2010 and January 2012. Chest X-ray examinations and TSTs were performed on each subject, while healthy volunteers, who passed medical examinations in the same period, were recruited as controls. There were a total of 122 cases in this study, including 87 men and 35 women, with a mean age of 38 ± 15 years (range 18-67) and a body mass index > 18.5 kg/m^2^ (Table 1). The general inclusion criteria for pulmonary tuberculosis in this study was the presence of an *M. tuberculosis* infection, confirmed by the mycobacterial sputum culture method (BACTEC 960 method) prior to the first treatment. Five international units of *M. tuberculosis* purified protein derivative were used for skin tests and a skin induration with a diameter over 10 mm was considered a positive response, whereas no skin induration was considered a negative response. Exclusion criteria included immune diseases, diabetes or tumors, a pulmonary disease caused by non-tuberculosis mycobacteria, multi-drug resistance determined by drug susceptibility testing, and HIV-positive status. The pulmonary tuberculosis subjects who met the inclusion criteria were divided into two groups based on the TST results. The first group consisted of 39 patients with anergic pulmonary tuberculosis (negative tuberculosis skin test results), including 29 men and 10 women, with a mean age of 39 ± 17 years. The second group consisted of 43 pulmonary tuberculosis patients with positive skin test results, including 28 men and 15 women, with a mean age of 37 ± 15 years. The control group consisted of 40 healthy individuals with positive skin test results, but with no abnormal chest X-ray findings, no history of tuberculosis, serious heart, liver, or kidney diseases, no history of allergic diseases and no history of taking any glucocorticoid and other immunosuppressive agents. This group included 30 men and 10 women, with a mean age of 40 ± 15 years.

**Table 1:** Basic information about the participants.

	Anergic Tuberculosis	TST-positive Tuberculosis	Control	Value	*P*
Age	39±17	43±15	40±15	*F*=0.515	0.599
Gender(Numbers)				X^2^=1.250	0.535
Male (%)	29 (74.4)	28 (65.1)	30 (75)		
Female (%)	10 (25.6)	15 (34.9)	10 (25)		
BMI(kg/m^2^)	21.2±3.4	21.9±5.8	22.1±2.5	*F*=1.603	0.194
Shanghai Resident (Numbers)	28	30	25	X^2^ = 0,876	0.645
With Stable Jobs (Numbers)	31	36	35	X^2^=0.926	0.629
With smoking history (Numbers)	21	22	30	X^2^=5.755	0.056

### Criteria for lesion severity scores

**Figure 1:**
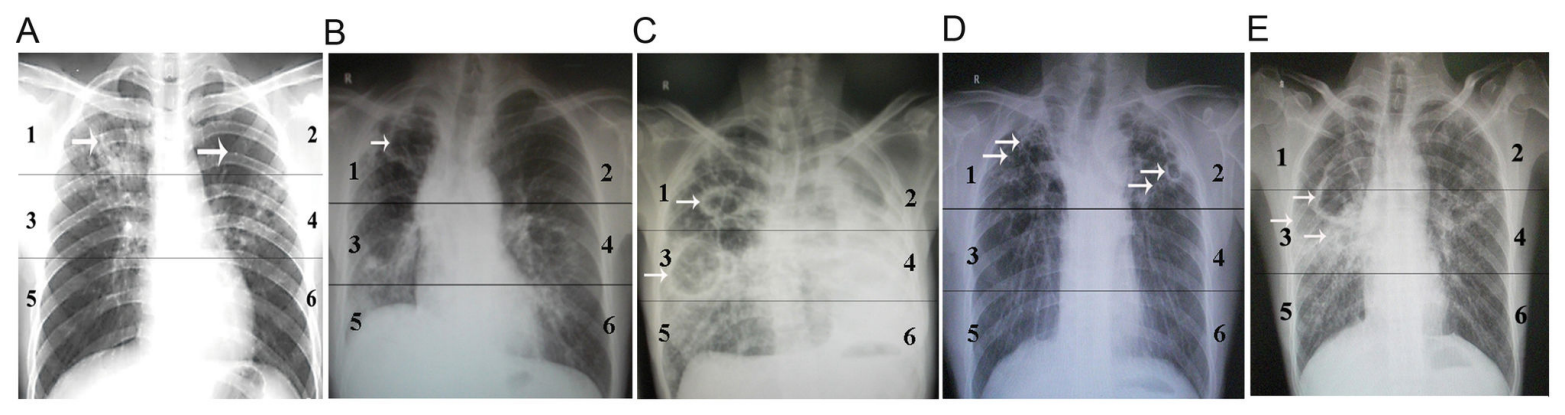
X-Ray images for lesion severity scoring. The white arrows indicate the lesions and cavities. A: Field 1, ≥50% of area affected = score of 2; Field 2, <50% of area affected = score of 1, B: Field 1, single cavity, <2cm diameter = score of 0.25, C: Field 1, single cavity, 2-4cm diameter = score of 0.5; Field 3, single cavity, >4cm diameter = score of 1, D: Field 1, multiple cavities, largest <2cm diameter = score of 0.5; Field 2, multiple cavities, largest 2-4cm diameter = score of 1, E: Field 3, multiple cavities, largest >4cm diameter = score of 2.

The chest X-rays of the tuberculosis patients were divided into six lung fields (Figure 1). The severity of the lung lesion was scored based on (a) the range of lung field foci and (b) the number/size of cavities (Table 2, Figure 1). The final lesion severity score was the sum of the scores of the six lung fields (every lung field = a+b of Table 2) and was ranked as follows: ≤ 2.5 as mild, 2.5-6 as moderate, and ≥ 6 points as severe (Table 3). All participants signed written informed consent forms and this study was approved by the Ethics Committee of the Shanghai Pulmonary Hospital.

**Table 2:** The criteria for lesion severity scores.

Disease (a)	Score
No disease	0
<50% of area affected	1
≥50% of area affected	2
Cavitation (b)	Score
No cavitation	0
Single cavity, <2cm diameter	0.25
Single cavity, 2-4cm diameter	0.5
Single cavity, >4cm diameter	1.0
Multiple cavities, largest <2cm diameter	0.5
Multiple cavities, largest 2-4cm diameter	1.0
Multiple cavities, largest >4cm diameter	2.0

**Table 3:** Number of patients with each severity score in the anergic and TST-positive groups.

Category	Anergic patients	TST positive patients
Mild (score ≤ 2.5)	8	18
Moderate(score 2.5-6)	8	11
Severe(score ≥ 6)	23	14

The final lesion severity score was the sum of the scores of the six lung fields (every lung field = a+b of Table 1, Figure 1) and ranked as follows: ≤ 2.5 as mild, 2.5-6 as moderate, and ≥ 6 as severe.

### Methods

#### Specimens

Prior to any anti-tuberculosis treatment, bronchoscopies were performed on tuberculosis patients under general or local anesthesia. A BF-F260 electronic bronchoscope (Olympus, Japan) was used for this procedure, and bronchi that showed severe lesions or cavities in the chest radiograph were rinsed with 100 ml saline; 20 ml of the resulting bronchoalveolar lavage fluid (BALF) was saved for further examination. In addition, 2 ml anti-coagulated venous blood was collected from each subject.

#### Flow cytometry

**Figure 2:**
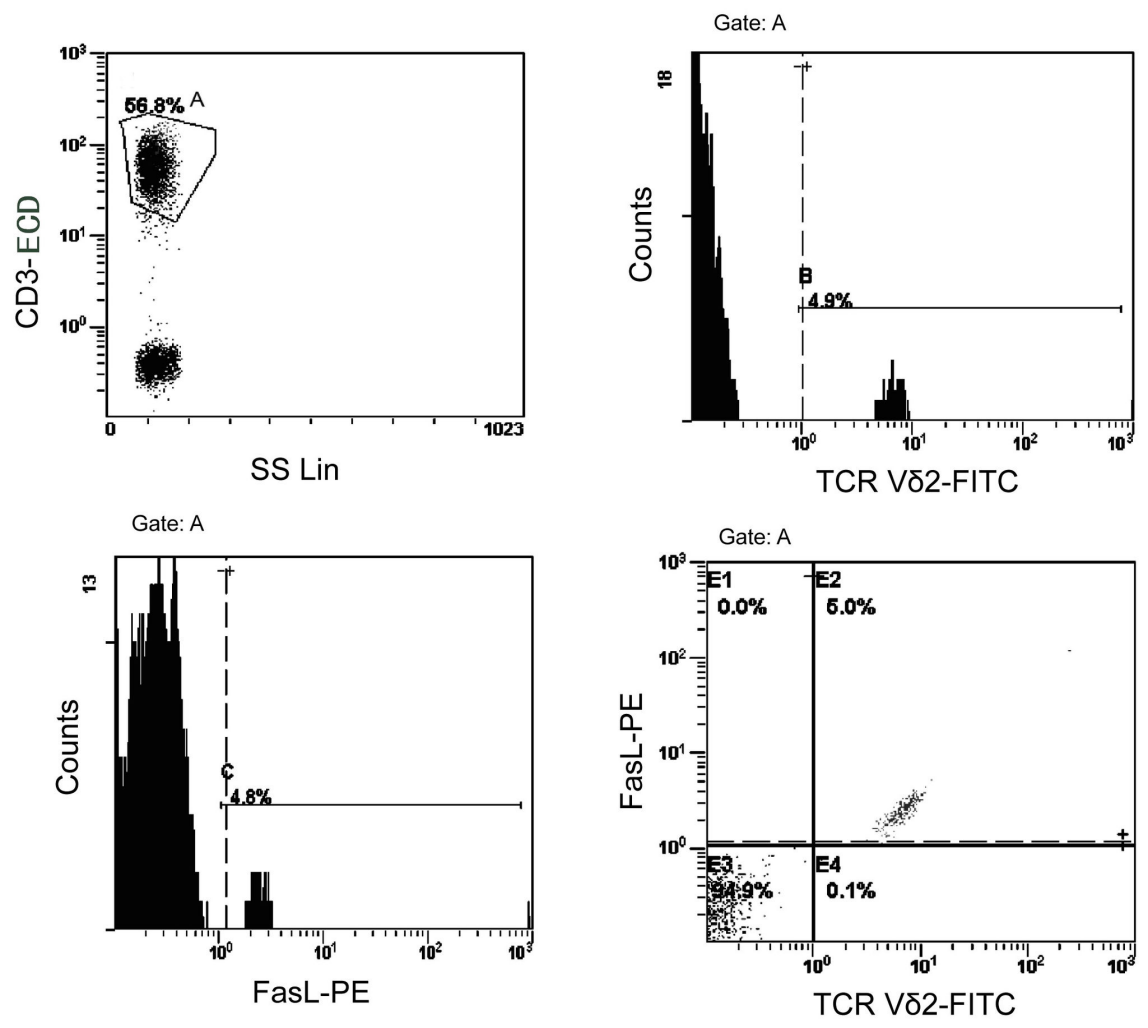
Flow cytometry gating strategy of Vδ2^+^ T cells and FasL expressing Vδ2^+^ T cells.

100 µl samples of anticoagulated blood from all three groups (anergic tuberculosis patients, TST-positive tuberculosis patients and healthy controls) as well as 5 ml samples of BALF from the patients with anergic tuberculosis and TST-positive tuberculosis were analyzed with FITC-TCR Vδ2^+^ antibodies (BD Bioscience). 10 µl of Phycoerythrin (PE)-FasL and CD3-Phycoerythrin-Texas red (CD3-ECD) antibodies (BD Bioscience) was added into the whole blood samples, which were then incubated at room temperature for 30 minutes in the dark. 1 ml red blood cell lysis buffer was added, and cells were incubated at room temperature in the dark for an additional 15-20 minutes. After vortexing, the suspensions were centrifuged at 1400 rpm for 5 minutes and the supernatant was discarded. The remaining cells were washed once with PBS and then resuspended in 400 µl PBS. Lymphocyte populations were gated based on the forward and side scatter lights (Beckman Coulter Cytomics FC500 flow cytometer (Beckman Coulter, Inc., USA) (Figure 2). The Vδ2^+^ T cells as a percentage of total lymphocytes and FasL expression levels of Vδ2^+^ T cells in the three groups of subjects were analyzed. The flow analysis acquisition equipment was the CXP Cytometer and the analysis software was CXP 2.2 Analysis.

#### Cytokines

For each γ- IFN, IL-2, IL-4, IL-6 and IL-10 quantification via ELISA (R&D Systems, Minneapolis, MN, USA), 200 µl of peripheral blood was used.

#### Statistical Analyses

The data are presented as mean (x) ± standard deviations (SD). The statistical software SPSS15.0 was used for analysis. Mean comparisons between groups were performed by using Student’s t test or ANOVA. Comparisons between paired groups were performed using paired t tests for two groups as well as SNK and LSD tests for multiple groups. The distribution analysis was performed by using Pearson’s X^2^ test. P values <0.05 were considered statistically significant.

## Results

### Demographic profiles of the three patient groups

There were no statistical differences among the three groups of subjects in terms of age, gender ratio, and body mass index, although the total number of men was 1.9 times that of the women. Therefore, the three groups were considered demographically comparable (Table 1).

### Correlation between lesion severity scores and Vδ2^+^ T cell percentage in the peripheral blood of the two tuberculosis patient groups

Based on the lesion severity scores determined by chest x-rays (≤ 2.5 ranked as mild, 2.5-6 as moderate, ≥6 as severe) of either the anergic tuberculosis or TST-positive tuberculosis patients, we found that 59% of anergic tuberculosis patients had “severe” lesions, and in these patients, the average Vδ2^+^ T cell percentage in the peripheral blood was 2.2 ± 1.2%; 20.5% of the anergic tuberculosis patients had “mild” lesions, and in these patients, the average Vδ2^+^ T cell percentage in the peripheral blood was 14.2 ± 12.0%. The percentage of TST-positive tuberculosis patients who had “severe” lesions was 32.6% and the corresponding Vδ2^+^ T cell percentage in the peripheral blood was 2.3 ± 0.8%. The percentage of TST-positive tuberculosis patients with a severity score of “mild” was 41.9%, which was higher than the percentages of patients with either “moderate” or “severe” scores, and in these patients with “mild” lesions, the percentage of peripheral blood Vδ2^+^ T cells was 14.0 ± 6.4% (X^2^=5.763, P=0.016) (Table 2, Table 3 and Table 4). All tuberculosis patients were divided into mild, moderate and severe subgroups based on chest radiograph scores. In the mild category the Vδ2^+^ T cell percentage in the peripheral blood was 14.2 ± 8.4%; the percentage was 6.0 ± 2.6% in the moderate category and 2.3 ± 1.1% in the severe category. A mean percentage value comparison among the three groups showed statistically significant differences (F = 45.149, P = 45.149). The more severe the lesions were, the lower were the concentrations of Vδ2^+^ T cells in the peripheral blood (Table 4). In summary, a high lesion severity score was correlated with a decreased Vδ2^+^ T cell percentage in the peripheral blood, a trend found in both anergic and TST-positive tuberculosis patients.

**Table 4:** Correlation between lesion severity scores and peripheral blood Vδ2^+^ T cell percentages.

Groups	Total #		Anergic tuberculosis patients		TST-positive tuberculosis patients	
	N (%)	Vδ2+T	N (%)	Vδ2+T	N (%)	Vδ2+T
Mild^a^	26 (31.7)	14.2±8.4	8 (20.5)	14.2±12.0	18 (41.9)	14.0±6.4
Moderate^b^	19 (23.2)	6.0±2.6	8 (20.5)	4.0±1.7	11 (25.6)	6.9±2.0
Severe^a^	37 (45.1)	2.3±1.1	23 (59)	2.2±1.2	14 (32.6)	2.3±0.8

a. Significantly different between two groups*, P<0.05*; b. Not significantly different between two groups, *P>0*.*05*.

### Vδ2^+^ T cell percentages and FasL expression levels in the peripheral blood and bronchoalveolar lavage fluid of anergic and TST-positive tuberculosis patients

#### Peripheral Blood

**Figure 3:**
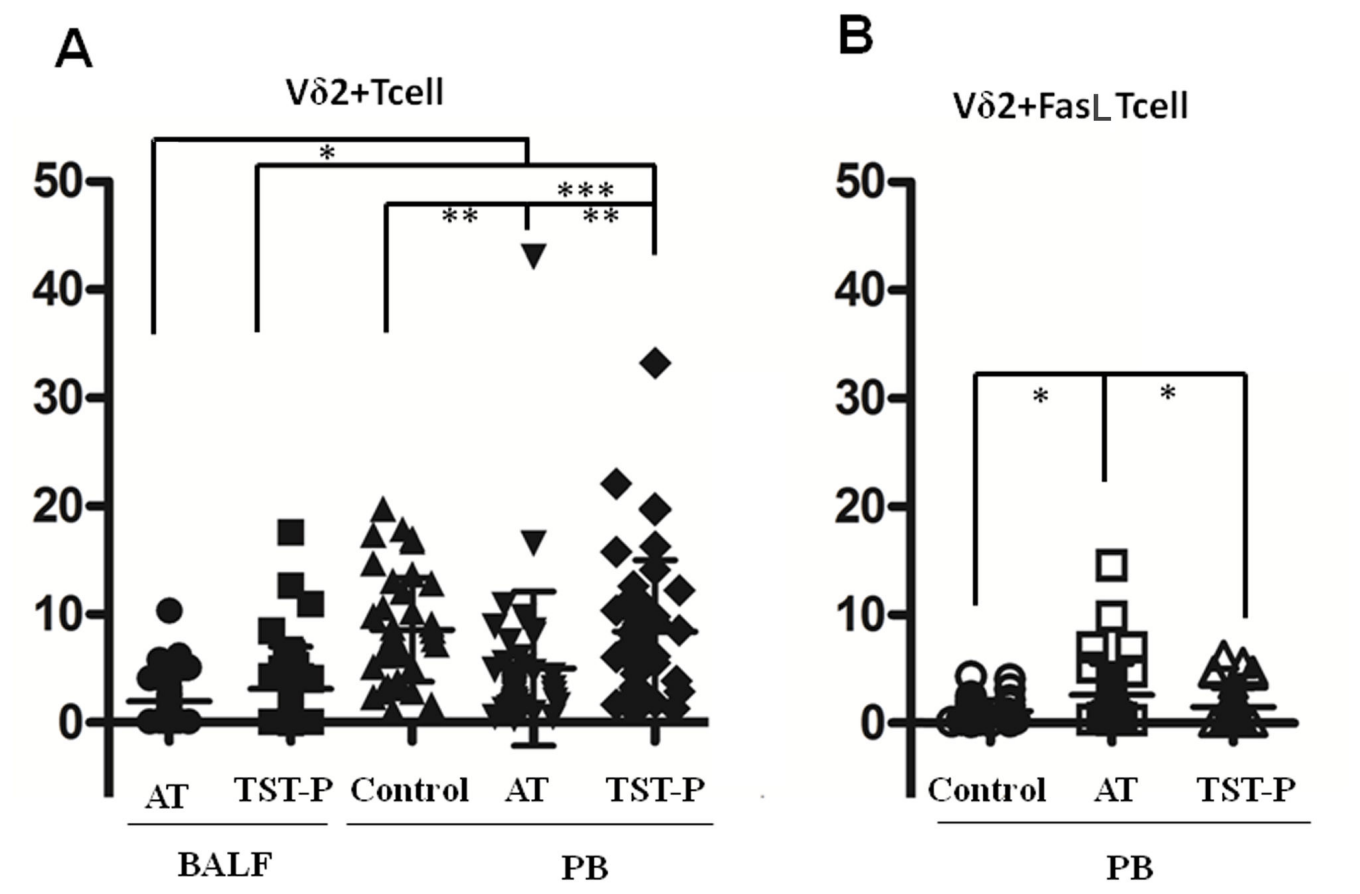
Vδ2^+^ T cell and FasL expressing Vδ2^+^ T cell percentages in peripheral blood and BALF of anergic tuberculosis patients (AT) and TST positive patients (TST-P). (A) Comparison of Vδ2^+^ T cell percentages in Peripheral Blood and BALF. (B) Comparison of FasL expressing Vδ2^+^ T cell percentages in peripheral blood. * P <0.05, **P<0.01, ***P<0.001.

The peripheral blood Vδ2^+^ T cell percentage (5.01 ± 7.11%) in anergic tuberculosis patients was significantly lower than in TST-positive tuberculosis patients (8.40 ± 6.64%) and healthy controls (8.57 ± 4.81%) (q = 2.448, 2.521, P = 0.016, 0.013). However, no statistically significant difference in peripheral blood Vδ2^+^ T cell percentage was identified between the TST-positive tuberculosis patients and the healthy controls (q = 0.118, P = 0.906) (Table 5, Figure 3A). Flow cytometry analyses showed that Vδ2^+^ T cell FasL expression levels in the peripheral blood of anergic tuberculosis patients (2.63 ± 2.84%) were significantly higher than in TST-positive tuberculosis patients (1.54 ± 1.70%) and healthy controls (1.13 ± 1.06%) (q = 2.440 and 3.326, P = 0.016 and 0.001). There was no statistically significant difference, however, between TST-positive tuberculosis patients and healthy controls in terms of FasL expression levels in peripheral blood Vδ2^+^ T cells (q = 0.951, P = 0.344) (Table 5, Figure 3B). In summary, anergic tuberculosis patients had lower Vδ2^+^ T cell percentages and more FasL positive Vδ2^+^ T cells in their peripheral blood compared to TST-positive tuberculosis patients and healthy controls.

**Table 5:** Percentage of Vδ2^+^ T cells and FasL expressing Vδ2^+^ T cells in peripheral blood and BALF.

Groups	Control		Anergic tuberculosis		TST-positive tuberculosis	
	BALF	PB	BALF	PB	BALF	PB
Vδ2+ T cell(%)	N/A	8.57±4.81	1.99±2.11^a^	5.01±7.11^b^	3.14±3.89^a^	8.40±6.64
Vδ2+FasL T cell(%)	N/A	1.13±1.06	N/A	2.63±2.84^b^	N/A	1.54±1.70

“PB”: Peripheral Blood; "N/A": not examined; a: was significantly lower than in peripheral blood, *P <0.05*; b: compared with peripheral blood of the other two groups, *P <0*.*05*.

### Bronchoalveolar lavage fluid (BALF)

The Vδ2^+^ T cell percentage (1.99 ± 2.11%) in the BALF of anergic tuberculosis patients was lower than in the BALF of TST-positive tuberculosis patients (3.14 ± 3.89%), but the difference was not statistically significant (t = 1.673, P = 0.099). However, the Vδ2^+^ T cell percentages in the BALF of the two groups of tuberculosis patients were both significantly lower than in the peripheral blood of the corresponding group (5.0 ± 7.1% for anergic patients and 8.4 ± 6.6% for TST-positive patients) (t = 2.575,6.645, P = 0.014,0.000) (Table 5, Figure 3A). Reduced Vδ2^+^ T cells in the BALF relative to the peripheral blood might be the result of activated T cell accumulation in the body. Taken together, these results suggest that FasL is predominantly expressed in activated T lymphocytes, and when the Fas signaling is blocked, activated T cells might accumulate unregulated in the body as a potential source for the development of autoimmune diseases.

### Comparison of the peripheral blood cytokine levels in the three groups

**Figure 4:**
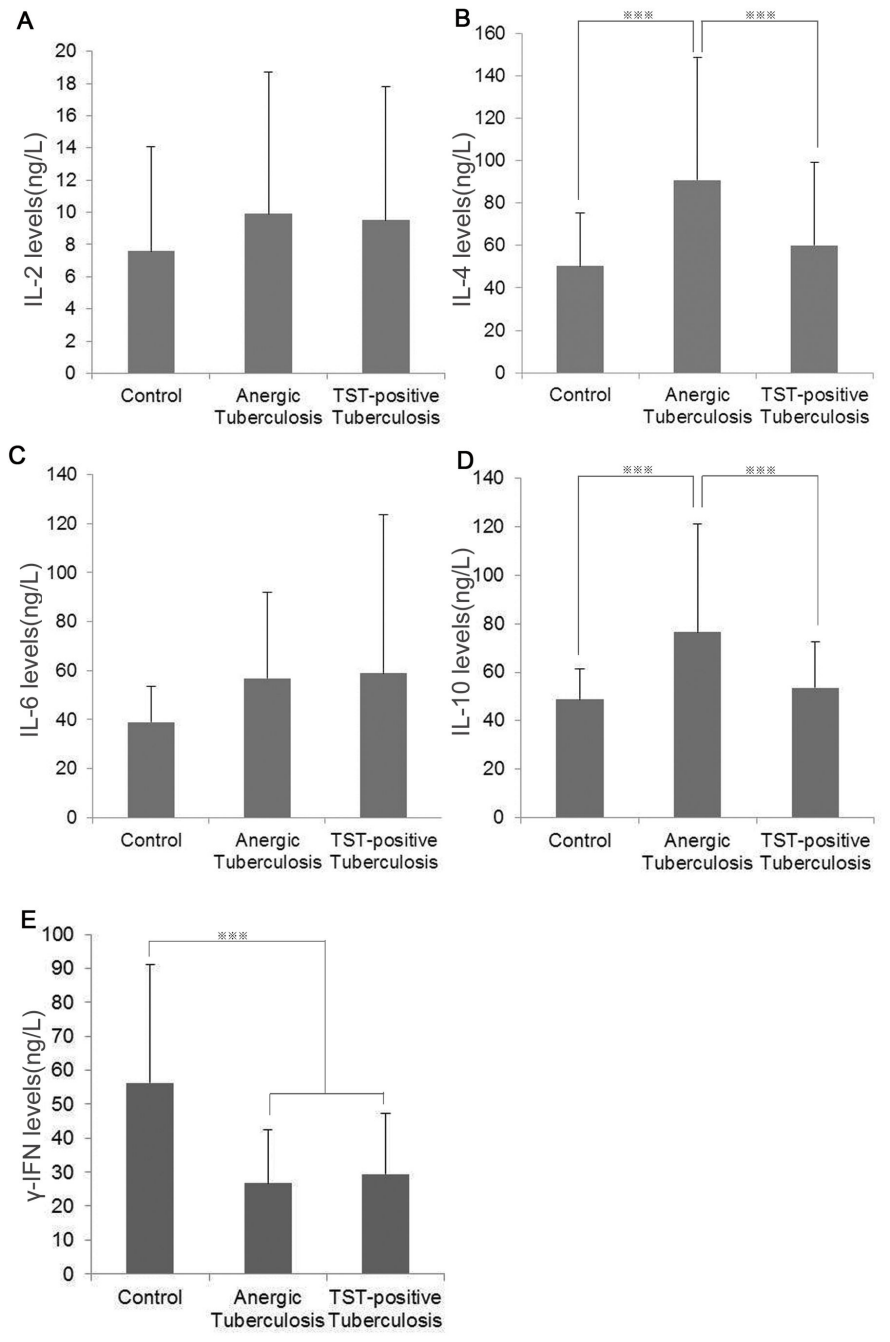
Comparisons of cytokine levels in the peripheral blood of anergic tuberculosis patients, TST-positive tuberculosis patients and TST positive healthy control subjects. ***P < 0.001.

The γ-IFN blood concentrations in both the anergic and TST-positive tuberculosis patients were significantly lower than in healthy controls (q = 5.424, 5.053, all P = 0.000). However, no statistically significant difference was identified between the two groups of tuberculosis patients in terms of γ-IFN values. The IL-4 and IL-10 blood concentrations in anergic tuberculosis patients [(91.0 ± 57.9) ng/L, (76.6 ± 44.8) ng/L] were significantly higher than in TST-positive tuberculosis patients [(60.1 ± 39.0) ng/L, (53.5 ± 18.9) ng/L] and in healthy controls [(50.4 ± 25.0) ng/L, (48.8 ± 12.6) ng/L] (q = 3.288, 3.646, P = 0.001, P = 0.000). TST-positive tuberculosis patients had higher IL-4 and IL-10 values than healthy controls, though the difference was not statistically significant (q = 1.043, 0.748, P = 0.299, 0.456). No statistically significant differences in IL-2 and IL-6 were identified among any of the groups (Figure 4).

## Discussion

Vδ2^+^ T cells are a type of intraepithelial lymphocytes that infiltrate the lymphatic systems of the mucosa. This subset of T cells accounts for less than 10% of all T cells in the peripheral blood of healthy individuals, but is predominant in organs such as the skin, reproductive tracts, tongue mucosa and respiratory epithelia. Since the respiratory epithelium mucosa and alveolar surface are the first places through which *M. tuberculosis* invades the host, Vδ2^+^ T cells might serve as a part of the first-line host immune defense against tuberculosis infections. It has been reported that reduction of Vδ2^+^ T cells in anergic tuberculosis patients is due to the inhibitory effects of regulatory T cells or dysregulation of Vδ2^+^ T cell functions [15–17]. In the present study, we found that the Vδ2^+^ T cells percentage in the peripheral blood of anergic tuberculosis patients was significantly lower than in TST-positive tuberculosis patients. In addition, the percentage of Vδ2^+^ T cells in the BALF of anergic patients was also very low; this suggests that a lack of Vδ2^+^ T cells in the peripheral blood of anergic tuberculosis patients was not caused by specific cell redistribution. Via *in vitro* co-culturing of *M. tuberculosis* antigens and γδ T cells, Li et al. found an induced Fas/FasL upregulation and subsequent Vδ2^+^ T cell apoptosis. In this study, the percentage of FasL-expressing Vδ2^+^ T cells in the peripheral blood of anergic tuberculosis patients was 1.7 times that of the TST-positive tuberculosis patients, suggesting that the lower Vδ2^+^ T cell concentration might be associated with enhanced FasL-mediated induced cell death. We observed very few Vδ2^+^ T cells in both the peripheral blood and BALF of anergic tuberculosis patients, a phenomenon that might be related to the severe clinical symptoms in this group and is in agreement with a previous report by Pinheiro et al., who suggested that peripheral γδ T cell reduction is strongly correlated with higher lesion severity in tuberculosis patients [18]. Furthermore, the presence of Vδ2^+^ T cells in the BALF in this study confirmed that alveolar macrophages infected with *M. tuberculosis* can become antigen-presenting cells and thus induce the activation of Vδ2^+^ T cells [19]. However, there was no observed increase in Vδ2^+^ T cell percentages in the BALF of tuberculosis patients, with or without positive skin test results; this requires further investigation. It is known that the activation of Vδ2^+^ T cells induces the secretion of a variety of cytokines, thereby both positively and negatively regulating immune responses. On one hand, Vδ2^+^ T cells can increase host immunity against infection either by secreting γ-IFN, which induces the apoptosis of infected cells, or by directly killing intracellular and extracellular *M. tuberculosis* through the production of granzyme or perforin. On the other hand, Vδ2^+^ T cells can also suppress host immunity against infections through the secretion of IL-4, IL-10 and other cytokines, thus avoiding overactive immune responses that may lead to the development of pathological lesions [20]. Consistent with a previous study by Thillai et al., our results revealed that the levels of IL-4 and IL-10 in the peripheral blood of tuberculosis patients were markedly higher than in healthy control participants [21]; however, in their measurements they did not distinguish between anergic and TST-positive tuberculosis patients. It has been shown that the level of IL-4 secretion is related to tuberculosis pathogenesis and host immune homeostasis [20]. In addition, IL-10 can induce the reduction of antigen presentation by down regulating the expression of co-stimulatory molecules in mononuclear cells and thus facilitate the rapid replication of lung *M. tuberculosis* in chronic tuberculosis patients [22]. Another study reported that elevated blood IL-4 levels in healthy individuals induced by contact with active tuberculosis patients for six months predicted the enhanced likelihood for these people to develop tuberculosis themselves [23]. In our study, we further determined the values of IL-4, IL-10 and other related cytokines specifically in anergic tuberculosis patients, which were significantly higher than in TST-positive tuberculosis patients and may be associated with the etiology of anergic tuberculosis. TST-positive and anergic tuberculosis patients had similar peripheral blood γ-IFN levels, both significantly lower than the γ-IFN levels in healthy controls; this might be due to the existence of other pathways regulating γ-IFN secretion, but further investigation is necessary to elucidate this. In summary, we suggest that the diminished number as well as functional impairment of Vδ2^+^ T cells in anergic pulmonary tuberculosis patients is associated with tuberculosis severity in these patients. In addition, we suggest that high expression of FasL triggers Vδ2^+^ T cell apoptosis, and increased IL-4 and IL-10 secretion induce an impairment of Vδ2^+^ T cell-mediated anti-tuberculosis immunity. Both factors might explain the severe clinical tuberculosis symptoms in anergic pulmonary tuberculosis patients.
